# Soluble VEGF receptor 1 (sFLT1) induces non-apoptotic death in ovarian and colorectal cancer cells

**DOI:** 10.1038/srep24853

**Published:** 2016-04-22

**Authors:** Tatsuya Miyake, Keiichi Kumasawa, Noriko Sato, Tsuyoshi Takiuchi, Hitomi Nakamura, Tadashi Kimura

**Affiliations:** 1 Department of Obstetrics and Gynecology, Osaka University Graduate School of Medicine, Suita 565-0871, Japan

## Abstract

Soluble Vascular Endothelial Growth Factor Receptor 1 (sVEGFR1/sFLT1) is an angiogenesis inhibitor that competes with angiogenic factors such as VEGF and Placental Growth Factor (PlGF). Imbalances of VEGF and sFLT1 levels can cause pathological conditions such as tumour growth or preeclampsia. We observed direct damage caused by sFLT1 in tumour cells. We exposed several kinds of cells derived from ovarian and colorectal cancers as well as HEK293T cells to sFLT1 in two ways, transfection and exogenous application. The cell morphology and an LDH assay revealed cytotoxicity. Additional experiments were performed to clarify how sFLT1 injured cells. In this study, non-apoptotic cell damage was found to be induced by sFLT1. Moreover, sFLT1 showed an anti-tumour effect in a mouse model of ovarian cancer. Our results suggest that sFLT1 has potential as a cancer therapeutic candidate.

In a previous study^1^,^2^,^3^,^4^, we developed a mouse model of preeclampsia by overexpressing placenta-specific human sFLT1 (hsFLT1). In these mice, only transduction of *sFLT1* decreased placental weight. To study the relationship between rapidly growing cells and sFLT1 overexpression, we have focused here on the effect of sFLT1 on highly proliferative tumour cells.

Vascular Endothelial Growth Factor (VEGF) and its soluble receptors are associated with endothelial dysfunction, vascular remodelling, and endothelial repair and regeneration mechanisms^2^,^5^,^6^,^7^. Soluble FLT1 is produced by a variety of tissues such as the placenta, endothelial cells and peripheral blood mononuclear cells^8^,^9^,^10^. Recently, several studies have demonstrated proliferative suppression by sFLT1 which caused apoptosis in an endothelial cell line^11^ and suppressed vascular development in the labyrinthine layer in a preeclampsia mouse model^4^. Moreover, systemic administration of AdV-*sFLT1* led to reduced tumour growth, tumour vascularity, and ascites formation in ovarian cancer xenografts^12^,^13^. A monoclonal antibody to VEGF, bevacizumab, is now clinically used as an antiangiogenic therapeutic for ovarian cancer, colorectal cancer and others^14^,^15^,^16^.

To the best of our knowledge, there is no literature clarifying the direct mechanism of cell injury by sFLT1. Previous reports^11^,^12^,^13^ have examined the secondary effects of anti-angiogenesis by sFLT1 *in vivo*. In the present study, we probed the mechanism of cell injury induced by sFLT1 by investigating the following four factors: 1) changes in cell morphology, 2) effects on cell proliferation signalling pathways, 3) effects on apoptosis, and 4) effects on non-apoptotic cell death. Furthermore, considering future therapies, we evaluated cytotoxicity in two ways: 1) transfection of LV-*sFLT1* into cells, and 2) exogenous administration of rVEGFR1 to culture media of four cell lines (HEK293T, SKOV3, HeyA8 and HT-29). Finally, we investigated the anti-tumour effect of exogenous rVEGFR1, endogenous sFLT1, and bevacizumab using mice transplanted with SKOV3 cells.

## Results

### Cell growth is restricted by sFLT1

**Figure 1:**
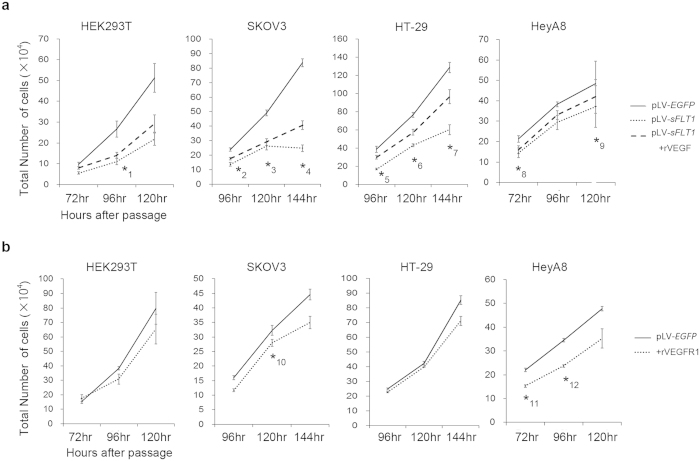
Soluble FLT1 has a suppressive effect against cell proliferation, and the effect is neutralized by VEGF. (**a**) The number of cells transfected with pLV-*EGFP* or pLV-*sFLT1* as well as with addition of VEGF. The number of cells was significantly lower in the sFLT1 group. *P < 0.05 compared to the pLV-*EGFP* group. (**b**) The number of cells transfected by pLV-*EGFP* as well as addition of rVEGFR1. In SKOV3 and HeyA8 cell lines, the cell numbers were significantly lower in the recombinant VEGFR1 groups compared to the control groups. *P < 0.05 compared to the pLV-*EGFP* group. P values are as follows; *1:P = 0.024, *2:P = 0.025, *3:P = 0.018, *4:P = 6.8 × 10^−4^, *5:P = 0.022, *6:P = 2.1 × 10^−3^, *7:P = 5.3 × 10^−3^, *8:P = 0.030, *9:P = 0.039, *10:P = 0.041, *11:P = 0.033, *12:P = 0.013.

To evaluate the effect of endogenous sFLT1 on cell proliferation, pLV-*sFLT1* or pLV-*EGFP* was transfected into the previously stated cell lines. We measured sFLT1 concentrations in the resulting culture media. These corresponded to the concentrations observed in women with preeclampsia or in normal pregnant women. pLV-*EGFP* was used as a control (Supplementary Table S1). Cell numbers were counted after transfection and did not differ significantly between the two groups 48 hours after passage, but following an additional 24 or 48 hours, there were significantly fewer cells in the sFLT1 treated group (P < 0.05) (Fig. 1a). Soluble FLT1 levels were confirmed to be higher in the pLV-*sFLT1*- transfected groups (Supplementary Table S2). A correlative tendency was also observed between low levels of VEGF and the suppression of cell proliferation (Supplementary Table S2). Next, to neutralize the activity of sFLT1, we added recombinant VEGF (rVEGF) to the cell lines which had been transfected with pLV-*sFLT1*, and the cell numbers recovered. Recombinant VEGF reversed the effect of sFLT1 (Fig. 1a). This not only showed that excessive VEGF did not accelerate cell growth (Supplementary Fig. S1) but it also confirmed the effect of sFLT1 transfection. We then investigated any cytotoxicity induced by sFLT1 at low serum levels; however, no significant changes were observed except at 0.1% serum and with *sFLT1* transfection into HeyA8 cells (Supplementary Fig. S2). Next, to evaluate future therapeutic applications, we treated cells exogenously with recombinant VEGF receptor 1 (rVEGFR1: equal to sFLT1 encoding protein). There were significantly fewer SKOV3 and HeyA8 cells in the rVEGFR1 groups compared to the controls (Fig. 1b). We also found that the compensatory effect of VEGF on cell number was reduced by sFLT1 expression (Fig. 1b).

### Cytotoxicity is induced by sFLT1

**Figure 2:**
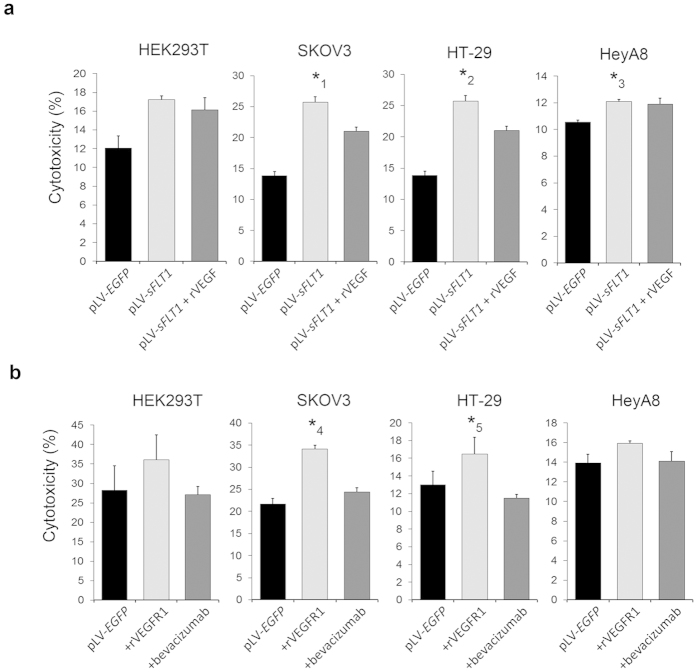
Both transfected and exogenously applied sFLT1 has cytotoxic activity. (**a**) Comparison of the LDH leakage assays after transfection with pLV-*EGFP* or pLV-*sFLT1*. In the 3 pLV-*sFLT1*-expressing tumour derived cell lines, the level of LDH release was significantly higher than for the pLV-*EGFP*-transfected group. With the addition of recombinant VEGF to the pLV-*sFLT1* groups, the levels of LDH release were restored to near those of the pLV-*EGFP* groups. Data are presented as a percentage of the control. *P < 0.05 compared to the pLV-*EGFP* group. (**b**) Comparison of the LDH leakage assay after treatment with recombinant VEGFR1 or bevacizumab. In SKOV3 and HT-29 cells, the level of LDH release was significantly higher, and in the other 2 cell lines, it was elevated but not significantly higher. *P < 0.05 compared to the pLV-*EGFP* group. P values are as follows; *1:P = 3.1 × 10^−3^, *2:P = 0.035, *3:P = 0.011, *4:P = 0.010, *5:P = 4.5 × 10^−4^.

We then examined the mechanism by which sFLT1 affected cell growth by quantifying lactate dehydrogenase (LDH) release into the medium. The pLV-*sFLT1*-expressing cells exhibited a higher level of LDH release compared to the control (pLV-*EGFP*-transfected) group in HEK293T, SKOV3, HeyA8, and HT-29 cells. When recombinant VEGF was added to the pLV-*sFLT1* groups, LDH release was restored to control levels, indicating that recombinant VEGF reversed the cytotoxic effect by neutralizing sFLT1 protein (Fig. 2a). We also examined the effect of exogenous sFLT1 treatment on LDH release. In SKOV3 and HT-29 cells, LDH release was significantly higher, and in the other cell lines, we observed a consistent albeit not a significant increase (Fig. 2b).

### sFLT1-induced cells appeared necrotic

**Figure 3:**
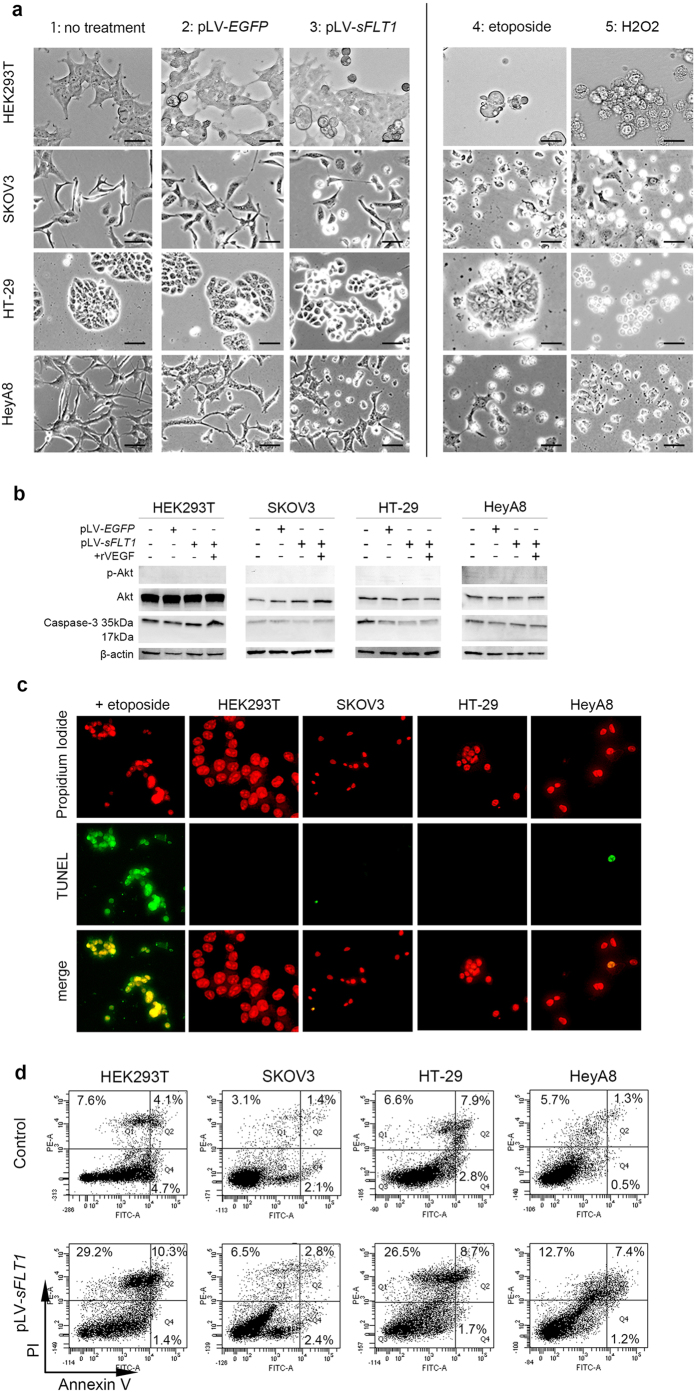
Both transfected and exogenously applied sFLT1/rVEGFR1 induce necrosis. (**a**) Morphology of HEK293T, SKOV3, HT-29, and HeyA8 cells, which were visualized under phase-contrast optics. **1**: No treatment. **2**: HEK293T, SKOV3, HT-29, and HeyA8 cells transfected pLV-*EGFP.* **3**: HEK293T, SKOV3, HT-29, and HeyA8 cells transfected with pLV-*sFLT1* were larger, and cell adhesion was disturbed. **4**: HEK293T, SKOV3, HT-29, and HeyA8 cells treated with 2.5 mM H_2_O_2_ to induce necrosis. The rupture of the cell membrane and swelling of the cells were observed. **5**: Treatment of HEK293T, SKOV3, HT-29, and HeyA8 cells treated with 25 μM etoposide to induce apoptosis showed apoptotic bodies and apoptotic cells with cytoplasmic aggregates. Scale bar, 10 μm. (**b**) Analysis for the presence of phosphorylated Akt and the cleaved subunit of caspase 3 by Western blotting. Neither phosphorylated Akt nor cleavage of caspase-3 was observed in the pLV-*sFLT1* group. (**c**) TUNEL staining was performed using a Dead End™ fluorometric TUNEL assay kit. Propidium iodide stains both apoptotic and non-apoptotic cells red. In HEK293T, SKOV3, HeyA8, and HT-29 cells, TUNEL-positive staining (green) was rarely observed in sFLT1-expressing cells. Scale bar, 20 μm. (**d**) Representative density plots showing the percentage of necrotic cells (area Q1) and early apoptotic cells (area Q4). Cells in Q2 include the combination of late apoptotic and necrotic populations. The cell death assessment was conducted by Annexin V / FACS analysis. The percentage of necrotic cells was larger in the group transfected with pLV-*sFLT1* compared to the non-transfected group.

It is widely accepted that *in vitro* treatment with H_2_O_2_ causes necrosis (fading nucleus, cell swelling, cell membrane rupture and release of cell contents), and treatment with etoposide causes apoptosis (nuclear condensation and vacuoles in cytoplasm)^17^,^18^. We evaluated the cell morphology of HEK293T, SKOV3, HT-29 and HeyA8 cells after pLV-*sFLT1* transfection and compared these cells with those treated by H_2_O_2_ or etoposide. We observed that some cells became larger, cell adhesion was disturbed, and cell membrane fragments floated in the culture medium, thus resembling H_2_O_2_ treated cells. These observations suggested that sFLT1 cytotoxic activity is caused by inducing necrosis (Fig. 3a).

### sFLT1 induced non-apoptotic effects

To investigate whether cell death was induced by apoptosis through a caspase pathway, cell lysates were analyzed for the presence of the cleaved subunit of caspase-3 or phosphorylated Akt by western blotting. No cleavage of caspase-3 nor phosphorylated Akt was detected, indicating that overexpression of sFLT1 did not affect the expression of caspase-3 (Fig. 3b). The identification of apoptotic cells using DNA fragmentation assays revealed the presence of a multitude of DNA strand breaks in transfected cells. In HEK293T, SKOV3, HeyA8, and HT-29, sFLT1-expressing cells were rarely TUNEL-positive, further indicating that sFLT1 has non-apoptotic effects (Fig. 3c).

The cells were analysed by FACS for annexin V expression and propidium iodide (PI) staining to determine the relative contribution of apoptosis and necrosis to cell death. Transfection of sFLT1 increased the percentage of necrotic cells compared to the non-transfected group (Fig. 3d).

### ERK and JNK phosphorylation was not downregulated by sFLT1

**Figure 4:**
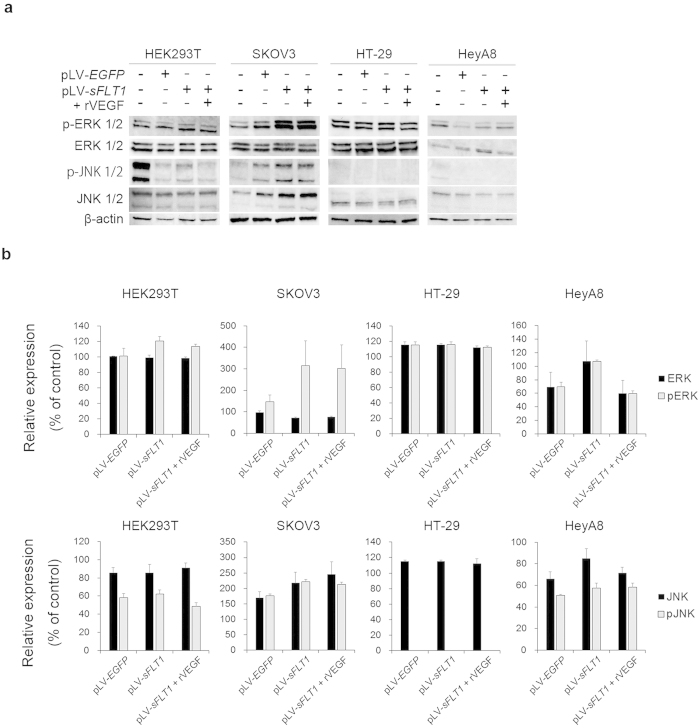
Overexpressed transfected sFLT1 does not have a major effect on cell proliferation signalling pathways or the cell cycle. (**a**) Western blot analysis of the MAPK-signalling pathway. (**b**) The analysis of activation of ERK1/2 and JNK1/2 in the MAPK-signalling pathway by Western blotting. Activation of ERK1/2 and JNK1/2 differed between cell lines, such that HEK293T, SKOV3 and HeyA8 cells showed an increase or no change after sFLT1 or rVEGF treatment, whereas activation of the MAPK pathway in HT29 cells was not affected. The data shown are expressed as percentages of the control and are presented as the mean ± S.E. values.

We next examined whether cell cycle signalling pathways were affected by sFLT1 overexpression in cultured cells. The activation of the extracellular signal regulated kinase 1/2 (ERK1/2) and c-Jun N-terminal kinase 1/2 (JNK1/2) differed between cell lines; HEK293T, SKOV3 and HeyA8 cells exhibited either increased or unchanged signalling after sFLT1 or rVEGF treatment, whereas HT29 cells were not affected. Overall we did not observe decreased activation of the ERK1/2 or JNK1/2 pathway by sFLT1 (Fig. 4a,b).

### Soluble FLT1 had an anti-tumour effect in mice transplanted with SKOV3 cells

To evaluate the anti-tumor effect of sFLT1 *in vivo*, we used nude mice transplanted with SKOV3 cells as an ovarian tumour model because this was the cell line most strongly affected by sFLT1 expression of those tested in our study. We also administered sFLT1 in two ways: transfection, in which we injected pLV-*sFLT1*-transfected SKOV3 cells into nude mice, and intraperitoneal administration of rVEGFR1 into tumour-bearing nude mice.

**Figure 5:**
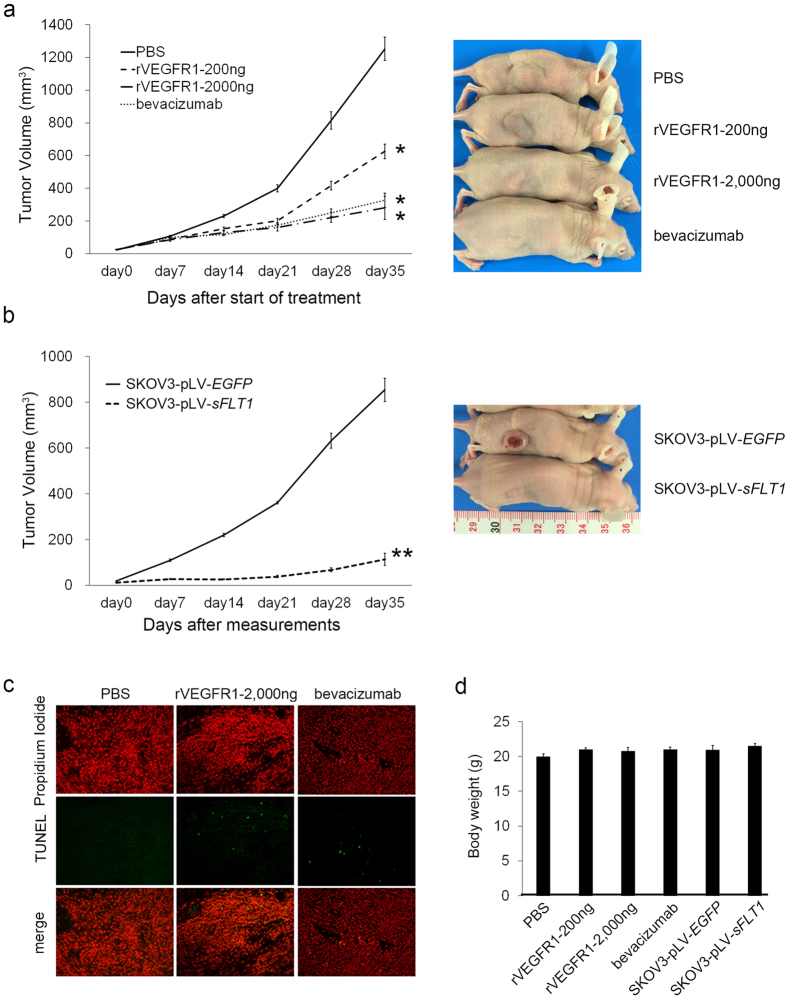
Soluble FLT1 shows an anti-tumour effect in mice injected with SKOV3 cells. Female nude mice were inoculated subcutaneously with SKOV3 cells. Treatments started when tumour volume exceeded 10 mm^3^, as described in Materials and Methods. (**a**) Anti-tumour activity of intraperitoneal administration of rVEGFR1. Recombinant VEGFR1-2,000 ng, rVEGFR1–200 ng and bevacizumab groups exhibited significantly smaller tumours compared to the PBS group (P = 0.0012, 0.0097, and 0.0038, respectively). Data points indicate the mean values ± S.E. of the tumour volume. Statistically significant differences are indicated by asterisks: *P < 0.05 significantly different from PBS treated mice. Body weight loss was not observed after treatment with rVEGFR1 or bevacizumab. (**b**) Anti-tumour activity of transfected sFLT1 in SKOV3 cells. The pLV-*sFLT1*-transfected group exhibited significantly smaller tumours compared to the pLV-*EGFP*-transfected group (P = 8.2 × 10^−4^). Data points indicate the mean values ± S.E. of tumour volume. Statistically significant differences are indicated by asterisks: **P < 0.05 significantly different from pLV-*EGFP* mice. (**c**) Representative images of apoptotic nuclear TUNEL staining using a Dead End™ fluorometric TUNEL assay kit. TUNEL-positive cells were visualized with green fluorescent protein at 20× magnification. Propidium iodide stains nucleus. TUNEL-positive staining was rarely observed in cells either exogenously rVEGFR1 or bevacizumab treated tumours. (**d**) Effect of the sFLT1 treatment on body weight. Each bar represents the mean ± S.E.

Thirty-five days after confirmation of tumour cell attachment, tumour size was markedly different (Fig. 5a,b). The tumour volumes (mean ± S.E.) were 280.1 ± 44.2 mm^3^ in the rVEGFR1–2,000 ng group, 625.1 ± 43.9 mm^3^ in the rVEGFR1–200 ng group, 327.3 ± 71.8 mm^3^ in the bevacizumab group and 1252.3 ± 71.5 mm^3^ in the PBS group. The recombinant VEGFR1–2,000 ng, rVEGFR1–200 ng and bevacizumab groups exhibited significantly smaller tumors compared with the PBS group (P = 0.0012, 0.0097, and 0.0038, respectively). Similarly, the tumour size in the pLV-*sFLT1*-transfected SKOV3 group (136.0 ± 26.3 mm^3^) was significantly smaller than that of the pLV-*EGFP*-transfected SKOV3 group (853.5 ± 51.7 mm^3^). Ki67 and CD31 staining was carried out on samples collected from tumors. CD31 staining decreased following rVEGFR1 or bevacizumab treatment (Supplementary Fig. S7a). Ki67 positive cells were also reduced by rVEGFR1 or bevacizumab (Supplementary Fig. S7b). TUNEL-positive staining was rarely observed in cells from exogenously treated rVEGFR1 tumours (Fig. 5c), indicating that non-apoptotic effects were also evident in the *in vivo* experiments.

We then examined possible side-effects of sFLT1. In pregnant women, high levels of sFLT1 have been associated with hypertension and proteinuria, known as preeclampsia. In mice, following intraperitoneal treatment with rVEGFR1, mean blood pressure was not elevated compared to the control group. In xenografted mice using HeyA8 cells transfected with pLV-sFLT1, the mean blood pressure was slightly elevated, though was not significantly higher than that of the pLV-*EGFP* group (Supplementary Table S3a).

With regard to urinary proteins, neither type of sFLT1 treatment affected the ratios of urinary albumin/urinary creatinine compared to the control group (Supplementary Table S3b).

## Discussion

In this study, we confirmed that sFLT1 induced cytotoxicity by measuring growth inhibition, LDH release and necrotic cellular morphology.

Caspase-3 expression and TUNEL staining in sFLT1-expressing cells indicated that the cellular damage was not caused by apoptosis. Furthermore, flow cytometry supported the conclusion that sFLT1 rather has necrotic effects on cells. Together, our findings show that sFLT1 induces necrotic rather than apoptotic cell damage.

Previous reports have suggested that sFLT1 has apoptotic effects on endothelial cells and induces tumor injury. Using *in vivo* experiments, the overall antitumor effects of sFLT1 have been described; however it was difficult to distinguish the direct effects of sFLT1 on the tumour cells from the tumor damage caused by its anti-angiogenic effects, prompting us instead to treat cell lines rather than tumors with sFLT1.

We examined the direct effects of sFLT1 on two ovarian cancer lines and one colorectal cancer cell line in addition to HEK293T cells. VEGF is a therapeutic target in ovarian and colorectal cancers, and bevacizumab is used commonly for these cancers. Bevacizumab was reported to be effective against SKOV3 cells, and HeyA8 has been injected into mice to generate an ovarian cancer model^19^,^20^. Furthermore, SKOV3 and HeyA8 cells have been reported to express moderate amounts of VEGFR1, whereas HT-29 cells have been reported to express lower levels of VEGFR1^21^,^22^,^23^. We used these cell lines to examine the mechanisms underlying the anti-tumour effects of sFLT1. Recombinant VEGFR1 had no effect on the HEK293T cell lines. In HEK293T cells, the concentration of sFLT1 in the supernatant of the rVEGFR1 group was approximately 5 times lower than that of the pLV-*sFLT1*-transfected group, which may be the reason why we failed to detect a significant difference, but only a tendency for cytotoxicity.

Our findings do not deny the apoptosis observed by other researchers. However, our study clarifies that non-apoptotic effects might also play an important role in the tumor cell damage caused by sFLT1. Similar to other reports, we tested low serum concentrations^24^,^25^. A cytotoxic effect of sFLT1 was not detected in the low serum media except in 0.1% FBS/HeyA8/pLV-*sFLT1* cells (Supplementary Fig. S2). These observations suggested that the cytotoxic effects of sFLT1 might require the presence of some additional elements present in serum.

Before implementing sFLT1 as a possible cancer therapeutic, it is important to examine possible side-effects. High levels of plasma sFLT1 can cause hypertension and elevated levels of urinary proteins^1^. Though the concentration in plasma might not be equivalent to that in the culture media of pLV-sFLT1 transfected cells, we noted that the sFLT1 concentration in the cell media was only about half that concentration of sFLT1 in human sera that causes preeclamptic symptoms. Soluble FLT1 is produced in physiological conditions and metabolized rapidly; moreover, excessive sFLT1 can be easily neutralized by VEGF. Therefore, the dose of sFLT1 for cancer therapy might be appropriately controlled with the minimization of negative side-effects.

For future therapeutic applications, we evaluated two types of sFLT1 administration—transfection of sFLT1 into tumor cells as well as exogenous treatment with sFLT1—and showed that both approaches effectively damaged tumour cells in a non-apoptotic way. Cancer progression requires three elements: growth, dissemination and metastasis. Angiogenesis is associated with growth and metastasis. Various studies have been performed and therapies have been developed based on these elements, such as bevacizumab treatment for tumour cells expressing VEGF. In this research, we directly elucidated the non-apoptotic effects of sFLT1, which combined with its anti-angiogenic properties would reinforce the inhibition of cancer growth and metastasis. Therefore, sFLT1 could be a strong therapeutic candidate for the treatment of ovarian and colorectal cancers in the future.

To assess cell lines derived from malignant tumours other than ovarian and colorectal cancers, we additionally used MCF-7 breast cancer cells and A549 lung cancer cells. Both MCF-7 and A549 cells were also affected by sFLT1 following both types of treatment (Supplementary Fig. S3). Therefore sFLT1 might have a cytotoxic effect on multiple cancers, and other cancers should to be tested with sFLT1 in the future. However, here we primarily investigated ovarian and colorectal cancers for possible VEGF-related therapy. Bevacizumab, a monoclonal antibody to VEGF, is widely used for ovarian and colorectal cancer treatment. For lung cancer, therapy with bevacizumab is restricted to only certain histological types, and for breast cancer, bevacizumab is used only for patients with metastases. Our results suggest therapeutic opportunities for a range of cancer types other than ovarian and colorectal cancers.

Based on the cytotoxic effect observed in the *in vitro* experiments, we also performed *in vivo* experiments, treating xenografted mice with sFLT1 using both types of sFLT1 administration. Intraperitoneal injection of rVEGFR1 was adopted as an exogenous method, as used in previous reports^26^. We tested different doses of rVEGFR1 and measured the level of serum sFLT1 (Supplementary Fig. S4). This declines abruptly, mimicking the trend seen in human pregnancy^2^, where after delivery, sFLT1 production from the placenta stops, and the level of serum sFLT1 decreases dramatically. Considering the rapid decline of exogenously applied sFLT1, we treated mice with sFLT1 three times per week intraperitoneally. In mice treated with 500 ng of sFLT1, the concentration of sFLT1 was as high as 344 pg/ml 2 hours after injection, and an anti-tumour effect was detected. Following 2,000 ng treatment with sFLT1, the anti-tumour effect was equivalent to that of bevacizumab. Moreover reduced CD31 staining of samples collected from tumors revealed less vascularity, and the reduced number of Ki67 positive cells indicated also less cell proliferation, thus also supporting the restriction of tumor growth induced by rVEGFR1 or sFLT1.

Excessive sFLT1 in pregnant women leads to hypertension and increased urinary protein, referred to as preeclampsia. In the present study, we measured blood pressure and urinary protein levels in our mouse tumor models. We observed that the mean blood pressure was not elevated by intraperitoneal treatment with sFLT1. Mean blood pressure tended to be higher after transplantation of sFLT1-expressing SKOV3 cells, an effect that might depend on the continuous secretion of sFLT1 although it was milder than previous observations^4^,^27^.

Urinary protein was evaluated using the urinary albumin/urinary creatinine ratio^4^. Urinary protein did not increase following either type of treatment with sFLT1. Moreover, sFLT1 levels decreased dramatically after termination of treatment, suggesting that even if side-effects appear, they will diminish rapidly after the last administration of sFLT1 (Supplementary Fig. S4).

In this study, we have elucidated the cytotoxic and anti-tumour effects of sFLT1 *in vitro* and *in vivo*, and show that sFLT1 is a potentially potent therapeutic agent against cancers.

## Methods

### Reagents and cell lines

Recombinant Human VEGF 165 (rVEGF) and recombinant Human VEGF R1 (rVEGFR1) (27–328 amino acids corresponding to the extracellular region of cell-surface FLT1) was purchased from R&D Systems. Bevacizumab was provided by Chugai Pharmaceutical Co. Ltd, Tokyo, Japan.

HEK293T human embryonic kidney cells were obtained from Riken Cell Bank, and SKOV3 human ovarian adenocarcinoma cells and HT-29 human colon adenocarcinoma cells were obtained from the American Type Culture Collection (ATCC). HeyA8 cells were kindly provided by Dr. Kenjiro Sawada (Osaka University, Japan). HEK293T and HeyA8 cells were cultured in DMEM (Invitrogen, Yokohama, Japan), and SKOV3 and HT-29 cells were cultured in McCoy’s 5a medium (Invitrogen). All of the media were supplemented with 10% fetal bovine serum, penicillin (100 IU/ml), and streptomycin (100 μg/ml). The cells were maintained in a humidified incubator at 37 °C in a 5% CO_2_ environment.

### Transient transfection

Plasmids encoding the lentiviral vectors for enhanced green fluorescent protein (pLV-*EGFP*) and soluble FLT1 were provided by the Genome Information Research Centre, Research Institute for Microbial Diseases, Osaka University, Japan. Cells were seeded in 6-well plates at densities of 2 × 10^4^ (HEK293T, HT-29 and HeyA8) or 4 × 10^4^ (SKOV3) cells in 2 ml culture medium per well and allowed to attach for 24 hours. The cells were transfected with 50 ng of pLV-*sFLT1* or pLV-*EGFP* as the control using Lipofectamine 2000 (Invitrogen). After transfection, recombinant VEGF (R&D systems, Minneapolis, Japan), rVEGFR1 (R&D systems) or bevacizumab (Chugai) was added to the culture medium and fresh medium was replaced every 3 days.

### Cell counting

The cell number was evaluated to determine the effect of sFLT1 transfection. HEK293T and HeyA8 cells were counted on days 3–5 days after passage, and SKOV3 and HT-29 cells were counted on days 4–6 after passage. Ten microliters of cell suspension were analysed using a haemocytometer.

### Lactate Dehydrogenase (LDH) release assay

Culture supernatants were collected, centrifuged at 200 g for 5 min, and transferred to new tubes. Lactate dehydrogenase (LDH) activity was measured using an LDH cytotoxicity detection kit (TaKaRa BIO Inc., Kusatsu, Japan). The percentage of LDH release was calculated using the following formula: percentage of release = 100 × (experimental LDH release − spontaneous LDH release)/(maximal LDH release − spontaneous LDH release). To determine the maximal LDH release, cells were treated with 1% Triton X-100.

### Western blot analysis

Cells were plated onto 6-well plates and allowed to attach for 24 hours. Then, pLV-*sFLT1* or pLV-*EGFP* was transfected into cells with/without recombinant VEGF (R&D systems) as described above. After 96 hours, the cells were washed twice with ice-cold phosphate-buffered saline (PBS) and lysed in lysis buffer supplemented with Protease Inhibitor Cocktail and Phosphatase Inhibitor Cocktail (Nakalai Tesque, Kyoto, Japan). The lysates were centrifuged at 3000 g at 4 °C, and the protein concentration was determined using a Protein Assay Kit (Bio-Rad, Hercules, Japan). Cell lysates (10 μg) were electrically separated using 7.5% SDS-PAGE and transferred to nitrocellulose membranes. After the membranes were blocked, they were incubated with antibodies against ERK, phospho-ERK, JNK, phospho-JNK, Akt, phospho-Akt, and Caspase 3 (Cell Signaling Technology, Tokyo, Japan) and then with a secondary horseradish peroxidase-conjugated IgG. The proteins were visualized with enhanced chemiluminescence (Perkin Elmer, Waltham, USA) according to the manufacturer’s protocol.

### Terminal deoxynucleotidyl transferase-mediated dUTP nick-end labelling (TUNEL) staining

Cells were seeded on a chamber slide (Thermo Scientific, Yokohama, Japan) at 2 × 10^4^ cells per 2 ml of culture medium, and pLV-*sFLT1* was transfected. After 48 hours, TUNEL staining was performed to detect *in situ* DNA fragmentation as a marker of apoptosis using the DeadEnd Fluorometric TUNEL system (Promega Corp, Fitchburg, USA). The cells were fixed in 4% paraformaldehyde for 15 min, rinsed with PBS and incubated in the equilibration buffer for 10 min at room temperature. The fixed cells were incubated with digoxigenin-conjugated dUTP in a recombinant terminal deoxynucleotide transferase (rTdT)-catalysed reaction for 1 hour at 37 °C in a humidified atmosphere and then immersed in 2 × SSC buffer for 15 min at room temperature to terminate the reaction. After washing with PBS, cells were incubated with 1 mg/ml propidium iodide (Wako, Osaka, Japan) solution for 30 min at 37 °C in the dark. Apoptotic cells were photographed using a fluorescence microscope (KEYENCE Corporation, Osaka, Japan).

### Apoptosis & necrosis assay

Cells were seeded in 6 cm dishes and allowed to attach for 24 hours. After transfection with pLV-*sFLT1* (120 hours), the cells were stained with 5 μl annexin-V-FITC and 5 μl propidium iodide by using Apoptosis and Necrosis Quantification Kit (Biotium, Inc, Fremont, USA) and analysed by flow cytometry (Becton-Dickinson, Franklin Lakes, USA).

### Therapeutic ovarian cancer model

All of the procedures involving animals and their care were approved by the Institutional Animal Care and Usage Committee of Osaka University in accordance with Japanese governmental guidelines for animal experiments. Female nude mice were obtained at 7 weeks of age and quarantined at least 1 week prior to the study. SKOV3 cells (5 × 10^6^ cells/mouse) in 0.2 ml PBS were injected subcutaneously into the right flank. Treatments were initiated when the tumour volume exceeded 10 mm^3^. Sixteen nude mice were assigned to four treatment groups, each containing four mice. The first group was treated with PBS twice weekly for 4 weeks as a control group. The second group was treated with 100 μg bevacizumab twice weekly for 4 weeks. The third group was treated with 200 ng rVEGFR1 three times per week for 4 weeks, and the fourth group was treated with 2,000 ng rVEGFR1 three times per week for 4 weeks. Bevacizumab and rVEGFR1 were administered intraperitoneally (i.p.).

To test the effect of endogenous overexpression of sFLT1, we prepared pLV-*sFLT1* or pLV-*EGFP* transfected calls. In brief SKOV3 cells were seeded in 10 cm plates at densities of 2.4 × 10^5^ in 10 ml of culture medium and allowed to attach for 24 hours. The cells were transfected with 300 ng of plasmids using Lipofectamine 2000 (Invitrogen). Eight nude mice were divided into two groups, and SKOV3-pLV-*EGFP* or SKOV3-pLV-*sFLT1* cells (5 × 10^6^ cells/mouse) in 0.2 ml PBS were injected subcutaneously into the right flank and tumor size was measured.

Body weight, abdominal circumference, and tumour volume were measured weekly until 5 weeks after the initial treatment. At this time, all of the mice were sacrificed to assess toxicity.

### Blood and urine samples

Blood samples were allowed to clot and were centrifuged to prepare serum. The concentration of total hsFLT1 were measured with ELISA kits according to the manufacturer’s instructions (R&D Systems). Urine albumin and creatinine concentrations were measured using Fuji DRI-CHEM 3500 V and DRICHEM slides (Fujifilm Co, Ltd, Tokyo, Japan) (Skylight Biotech, Inc, Akita, Japan).

### Measurement of blood pressure

Blood pressure was measured using the tail-cuff method with a BP98A (Softron Co, Ltd, Tokyo, Japan). The mice were gently placed in a small cage without anaesthesia, and their blood pressures were measured after their behaviour, heart rates, and blood pressures were stabilized. After stabilization, both systolic and diastolic blood pressure was recorded at least five times and up to 10 times until the stabilization was broken. The mean of both the systolic and diastolic blood pressures measured as indicated above and were used for further statistical analysis.

### Statistical Analysis

Results are presented as the mean +/− standard error of the mean (S.E.). Significant differences were reported when the probability value was less than 0.05 (P < 0.05). The data were analysed using Student’s t-test, Chi square test or Fisher test using Sigma Stat 3.5.

## Additional Information

**How to cite this article**: Miyake, T. *et al.* Soluble VEGF receptor 1 (sFLT1) induces non-apoptotic death in ovarian and colorectal cancer cells. *Sci. Rep.* **6**, 24853; doi: 10.1038/srep24853 (2016).

## Supplementary Material

Supplementary Information
