# Supplementary material for: Soluble VEGF receptor 1 (sFLT1) induces non-apoptotic death in ovarian and colorectal cancer cells
Source: Sci Rep. 2016 Apr 22;6:24853. doi: 10.1038/srep24853 (PMC4840331; doi:10.1038/srep24853)
Supplement: Supplementary Information [file srep24853-s1.pdf]

TITLE: Soluble VEGF receptor 1 (sFLT1) induces non-apoptotic death in ovarian and colorectal cancer cells

AUTHORS: Tatsuya Miyake[1], Keiichi Kumasawa*[1], Noriko Sato[1], Tsuyoshi Takiuchi[1], Hitomi Nakamura[1], Tadashi Kimura[1]

AFFILIATIONS: [1]Department of Obstetrics and Gynecology, Osaka University Graduate School of Medicine, Suita 565-0871, Japan

Corresponding Author: Keiichi Kumasawa

E-mail: kumasawa@gyne.med.osaka-u.ac.jp

SI Methods

Reagents and cell lines. Recombinant Human VEGF 165 (rVEGF) and recombinant Human VEGFR1 (rVEGFR1) was purchased from R&D Systems. Bevacizumab was provided by Chugai Pharmaceutical Co. Ltd.HEK293T human embryonic kidney cells were obtained from Riken Cell Bank, and SKOV3 human ovarian adenocarcinoma cells as well as HT-29 human colon adenocarcinoma cells were obtained from the American Type Culture Collection (ATCC). HeyA8 cells were kindly provided by Dr. Kenjiro Sawada (Osaka University, Japan). MCF7 human breast adenocarcinoma cells were obtained from the Japanese Collection of Research Bioresources (JCRB; Osaka, Japan). A549 human lung carcinoma cells were kindly provided by Dr. Izumi Nagatomo and Dr. Haruhiro Hirata (Osaka University, Japan). HEK293T and HeyA8 cells were cultured in DMEM (Invitrogen), and SKOV3 and HT-29 cells were cultured in McCoy's 5a medium (Invitrogen). All media were supplemented with 10% fetal bovine serum, penicillin (100 IU/ml), and streptomycin (100μg/ml). MCF7 cells were cultured in Eagle's minimum essential medium with non-essential amino acids, 1 mM sodium pyruvate and 0.01 mg/ml insulin with 10% fetal bovine serum. A549 cells were cultured in DMEM (Invitrogen) with 10% fetal bovine serum, penicillin (100 IU/ml), and streptomycin (100μg/ml). Cells were maintained in a humidified incubator at 37℃ in a 5% CO_2 in air environment.

Transient transfection.

pLV-*EGFP* and pLV-*sFLT1* were provided by the Genome Information Research Centre in Microbial Disease Research Institute, Osaka University, Japan. Cells were seeded in 6-well plates at densities of 2×10^4 (HEK293T, HT-29 and HeyA8), 4×10^4 (SKOV3), 6×10^4 (MCF7) and 8×10^4 (A549) cells in 2mL of culture medium per well, respectively, and allowed to attach for 24 hours. The cells were transfected with 50 ng of either pLV-*sFLT1* or pLV-*EGFP* as control, using Lipofectamine 2000 (Invitrogen). After transfection, 800pg of rVEGF (R&D systems) or 2,200ng of rVEGFR1(R&D systems) or 500μg of bevacizumab (Chugai) were added into the culture medium.

Cell Counting.

Cell number was evaluated to determine the effect of sFLT1 transfection. In HEK293T and HeyA8, cells were counted for 3-5 days after passage, and in SKOV3 and HT-29, for 4-6 days after passage. MCF7 and A549 cells were counted 4-6 days after passage. Ten microliters of cell suspension was analyzed using a hemocytometer.

Low Serum Concentration

Cells were seeded in 6-well plates at densities of 2×10^4 (HEK293T, HT-29 and HeyA8) and 4×10^4 (SKOV3) cells in 2mL of culture medium per well, respectively, and allowed to attach for 24 hours. The cells were

transfected with 50 ng of either pLV-*sFLT1* or pLV-*EGFP* as control, using Lipofectamine 2000 (Invitrogen). After 24 hours, medium was removed and replaced with medium containing 1% or 0.1% fetal bovine serum. At the same time, 800pg rVEGF or 2,200pg rVEGFR1 or 500μg bevacizumab were added into the culture medium. Cells were counted for 4-6 days after passage. Ten microliters of cell suspension was analyzed using a hemocytometer. For the LHD cytotoxic assay, culture supernatants were collected 120 hours after passage. Then, culture supernatants were centrifuged at 200g for 5 min, and transferred to new tubes. Lactate dehydrogenase (LDH) activity was measured using an LDH cytotoxicity detection kit (TAKARA BIO Inc., Japan). The percentage of LDH release was calculated using the following formula: percentage of release = 100 × (experimental LDH release − spontaneous LDH release) / (maximal LDH release − spontaneous LDH release). To determine the maximal LDH release, cells were treated with 10% Triton X-100.

Cell Proliferation Assay.

Cellular DNA synthesis rates were determined by measuring bromodeoxyuridine (BrdU) incorporation with the commercial BrdU Cell Proliferation enzyme-linked immunosorbent assay (ELISA) kit (Roche Molecular Biochemicals, Mannheim, Germany). Cells were seeded in 96-well plates and allowed to attach for 24 hours. The cells were then transfected with pLV-*sFLT1* or pLV-*EGFP*. After transfection (48 hours), the cells were incubated for 3 hours with a BrdU-labeling solution (provided in the kit) containing 10 mmol/L BrdU. The assay was performed according to the manufacturer’s instructions. Absorbance values were measured at 450 nm (reference wavelength: 492nm) using an ELISA reader (SH-9000; Corona Electric Co, Japan). All the samples were run at least 5 times in the same experiment and repeated in 3 experiments.

ELISA.

Cells were seeded in 6-well plates at densities of 2×10^4 (HEK293T, HT-29 and HeyA8) and 4×10^4 (SKOV3) cells in 2mL of culture medium per well, respectively, and allowed to attach for 24 hours. In the endogenous treatment group, cells were transfected with pLV-*EGFP* or pLV-*sFLT1*, and in the exogenous treatment group, rVEGF (R&D systems), rVEGFR1 (R&D systems) or bevacizumab (Chugai) was added to the culture medium after transfection of pLV-*EGFP*, and fresh medium was replaced every 3day. In HEK293T and HeyA8 cells, culture supernatants were collected 96 hours after passage, and for SKOV3 and HT-29 cells, 120 hours after passage. We determined the concentrations using commercial ELISA kits (R&D Systems) and according to the manufacturer’s specifications.

Immunochemical analysis.

Ki-67: Tissues were fixed in 10% formalin solution for 24 hour and then were paraffin embedded, sectioned and rehydrated. Antigen retrieval on paraffin sections was performed by heating at 110°C for 15 min in 10 mM citrate buffer solution (pH 6.0) in an oil bath. Endogenous peroxidase was blocked by incubating the sections in

3.0% H_2O_2 for 30 min. After blocking nonspecific reactivity with goat serum for 10 min at room temperature, the sections were incubated overnight at 4°C with a primary antibody against Ki-67 (Novocastra, UK). The primary antibody was visualized using subsequent application of a secondary biotinylated antibody (Histofine SAB PO kit) and streptavidin-peroxidase (Histofine SAB PO kit). Immunostaining was developed using Envision DAB (Dako Ltd.) and the sections were counterstained with Mayer's haematoxylin. As a negative control, some sections were subjected to normal serum blocking with omission of the primary antibody.

CD31: Tissues were frozen without fixation and sectioned at 100 μm thickness with a cryostat. Endogenous peroxidase was blocked by incubating the sections in 3.0% H_2O_2 for 30 min. After blocking nonspecific reactivity with rabbit serum for 10 min at room temperature, the sections were incubated overnight at room temperature in a rat anti-mouse CD31 monoclonal antibody (BD PharMingen, USA) diluted 1:200, followed by 30 min in biotin-labelled rabbit secondary anti-rat IgG antibody (Dako, USA) diluted 1:600. Immunostaining was developed using Envision DAB (Dako, USA), and the sections were counterstained with Mayer's haematoxylin. As a negative control, some sections were subjected to normal serum blocking with omission of the primary antibody.

SI Figure Legend

Supplementary Figure S1 | Only excessive VEGF did not accelerate cell growth. To evaluate the effect of additional VEGF on cell proliferation, pLV-*EGFP* was transduced into HEK293T, SKOV3, HeyA8, and HT-29 cells, and at the same time as the transfection we added rVEGF into the culture media. The number of cells with or without additional rVEGF was not significantly different in HEK293T, SKOV3, HT-29, and HeyA8 cells.

Supplementary Figure S2 | In low serum concentration, there is no significant cytotoxic change caused by sFLT1. (**a**) The number of cells transfected by pLV-*EGFP* or pLV-*sFLT1* in medium with 1% FBS or 0.1% FBS, and with additional VEGF. There was no significant change in cell number for the sFLT1 group compared to the EGFP group. (**b**) The number of cells transfected by pLV-*EGFP* in medium with 1% FBS or 0.1% FBS, and with additional rVEGFR1. There was no significant change in cell number for the rVEGFR1 groups compared to the EGFP group. (**c**) Comparison of LDH leakage assay after transfection with pLV-*EGFP* or pLV-*sFLT1* in medium with 1% FBS or 0.1% FBS. In all 4 cell lines, there was no significant difference in the sFLT1 group compared to the EGFP group. (**d**) Comparison of LDH leakage assay after treatment with rVEGFR1 or bevacizumab in medium with 1% FBS or 0.1% FBS. In all 4 cell lines, there was no significant difference in rVEGFR1 group compared to the EGFP group.

Supplementary Figure S3 | Soluble FLT1 has a suppressive effect against cell proliferation in MCF-7 and A549 cells, and the effect is neutralized by VEGF. (**a**) The number of cells transfected with pLV-*EGFP* or pLV-*sFLT1*, and with additional VEGF. Cell number was significantly reduced in the sFLT1 group. *P<0.05 versus EGFP group. (**b**) The number of cells transfected by pLV-*EGFP*, and with additional rVEGFR1. In HeyA8 and SKOV3 cell lines, cell numbers were significantly lower in rVEGFR1 groups compared with control groups. *P<0.05 versus EGFP group. (**c**) Comparison of LDH leakage assay after transfection with pLV-*EGFP* or pLV-*sFLT1*. In MCF7 cells, the level of LDH release in pLV-*sFLT1* transfected group was significantly higher than that of the pLV-*EGFP* transfected group. And in A549 cells, we also found a similar tendency. With addition of rVEGF to the sFLT1 groups, the levels of LDH release were restored to values near to those of the EGFP groups. Data are presented as percentage of control. *P<0.05 versus EGFP group. (**d**) Comparison of LDH leakage assay after treatment with rVEGFR1 or bevacizumab. In A549 cells, the level of LDH release was significantly higher. Furthermore, rVEGF restored the level of LDH release. *P<0.05 versus EGFP group.

Supplementary Figure S4 | The level of serum sFLT1 decreased rapidly after intraperitoneal administration in mice. Blood samples were obtained at 2, 24 and 48 hours after intraperitoneal administration of 2,000ng or 500ng recombinant human VEGF receptor 1. Concentrations of total hsFLT1 were measured with an ELISA kit. The level of serum sFLT1 decreased abruptly.

Supplementary Figure S5 | Addition of bevacizumab did not affect cell growth. To evaluate the effect of additional bevacizumab on cell proliferation, pLV-*EGFP* was transfected into HEK293T, SKOV3, HeyA8, and HT-29 cells, and at the same time as the transfection, we added bevacizumab into the culture medium. The number of cells with or without additional bevacizumab was not significantly different in HEK293T, SKOV3, HT-29, and HeyA8 cells.

Supplementary Figure S6 | Cellular DNA synthesis rates were determined by measuring bromodeoxyuridine (BrdU) incorporation with the commercial BrdU Cell Proliferation enzyme-linked immunosorbent assay (ELISA) kit. (**a**) Cells were seeded in 96-well plates and allowed to attach for 24 hours. The cells were then transfected with pLV-*sFLT1* or pLV-*EGFP*, and treated with rVEGF. After transfection (48 hours), the cells were incubated for 3 hours with a BrdU-labeling solution. The assay was performed according to the manufacturer’s instructions. Absorbance values were measured at 450 nm (reference wavelength: 492nm) using an ELISA reader (SH-9000; Corona Electric Co). All samples were measured at least 5 times in the same experiment and repeated in 3 experiments. There was no difference of proliferation rate between pLV-*EGFP* and pLV-*sFLT1* groups for each cell line. (**b**) The cells were transfected with pLV-*EGFP*, and treated with rVEGFR1 or bevacizumab. There was no difference of proliferation rate between pLV-*EGFP* and rVEGFR1 groups in all cell lines.

Supplementary Figure S7 | Immunochemical analysis. (**a**) Representative images of CD31 staining. Quantification was determined by measuring the CD31-stained area with Image J software. CD31-positive, ring-like structures were considered as lumen. Scale bar, 100 µm. Data are shown as the means ± S.E. Student's t test was performed. Statistically significant differences are indicated by asterisks: *, $P < 0.05$ significantly different from PBS-treated mice; **, $P < 0.05$ significantly different from SKOV3-*EGFP* mice. (*1:P=0.016, *2:P=1.2×10^{-3},*3:P=5.8×10^{-4}, **4:P=2.5×10^{-3}) (**b**) Representative images of Ki-67 staining. Quantification was determined by counting five different fields per tumour, followed by averaging. Scale bar, 100 µm. Data are shown as the means ± S.E. Student's t test was performed. Statistically significant differences are indicated by asterisks: *, $P < 0.05$ significantly different from PBS treated mice; **, $P < 0.05$ significantly different from SKOV3-*EGFP* mice. (*5:P=9.1×10^{-4}, *6:P=3.3×10^{-6}, **7:P=2.2×10^{-3})

Supplementary Table S1 | The relationship between the concentration of sFLT1 in culture supernatants and the amounts of plasmid DNA. ELISAs. The relationship between the concentration of sFLT1 in culture supernatants and the amounts of plasmid DNA. Cells were seeded in 6-well plates at densities of 2×10^4 (HEK293T, HT-29 and HeyA8) and 4×10^4 (SKOV3) cells in 2mL of culture medium per well, respectively, and allowed to attach for 24 hours. Then, the cells were transfected with various concentrations of pLV-*sFLT1.* In HEK293T and HeyA8 cells, culture supernatants were collected 72 hours after transfection, and in SKOV3 and HT-29 cells, collected 96 hours after transfection. We determined the concentrations using commercial ELISA kits (R&D Systems) according to the manufacturer's specifications.

Supplementary Table S2 | The concentrations of sFLT1, VEGF and PIGF in supernatants of culture dishes. ELISAs. The concentrations of sFLT1, VEGF and PIGF of supernatants of culture dishes revealed a correlation between lower concentration of VEGF and fewer cell numbers. We determined the concentrations using commercial ELISA kits (R&D Systems) according to the manufacturer's specifications.

Supplementary Table S3 | Evaluation of side effects caused by sFLT1 in ovarian cancer model mice. (**a**) Systolic and diastolic blood pressure were measured at five weeks after initial treatment (n = 4). In intraperitoneal treatment experiments, the mean blood pressure of neither the rVEGFR1-2000ng nor the rVEGR1-200ng groups was elevated compared to the control group. On the other hand, with injection of transfected cells, the mean blood pressure of the sFLT1 group tended to be higher than that of the EGFP group. (P=0.16) (**b**) Urine albumin and creatinine concentrations were analyzed at five weeks after initial treatment. Student's t test was performed against the PBS group or the SKOV3-pLV-*EGFP* group as control.

The ratios of urinary albumin/urinary creatinine in the rVEFGR1 group or SKOV3-pLV-*sFLT1* group was similar to those in the control group.

Supplementary Figure S1

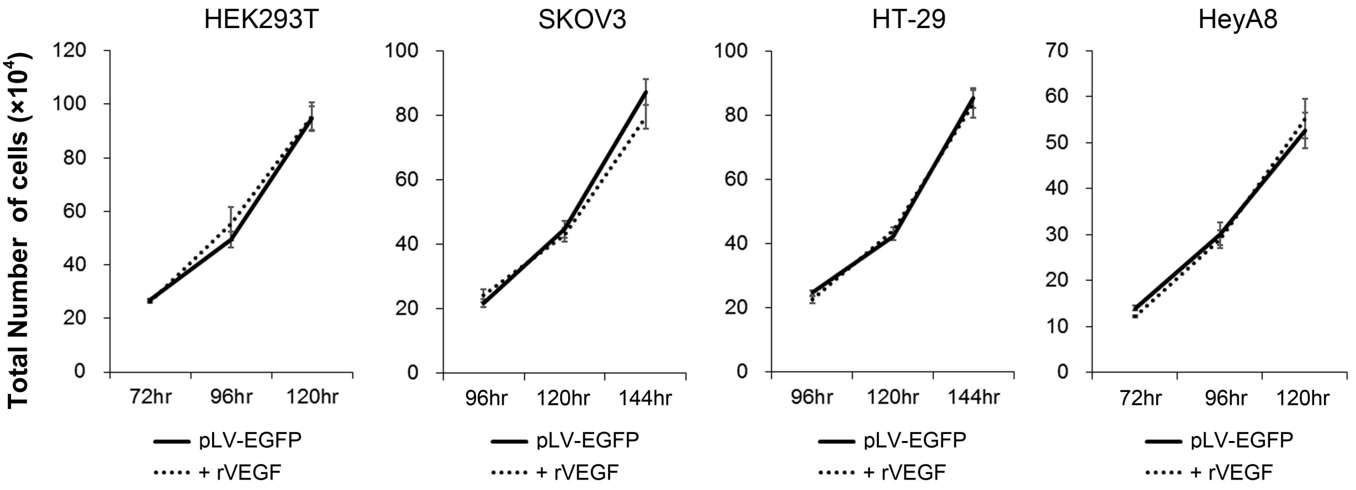

Supplementary figure S2

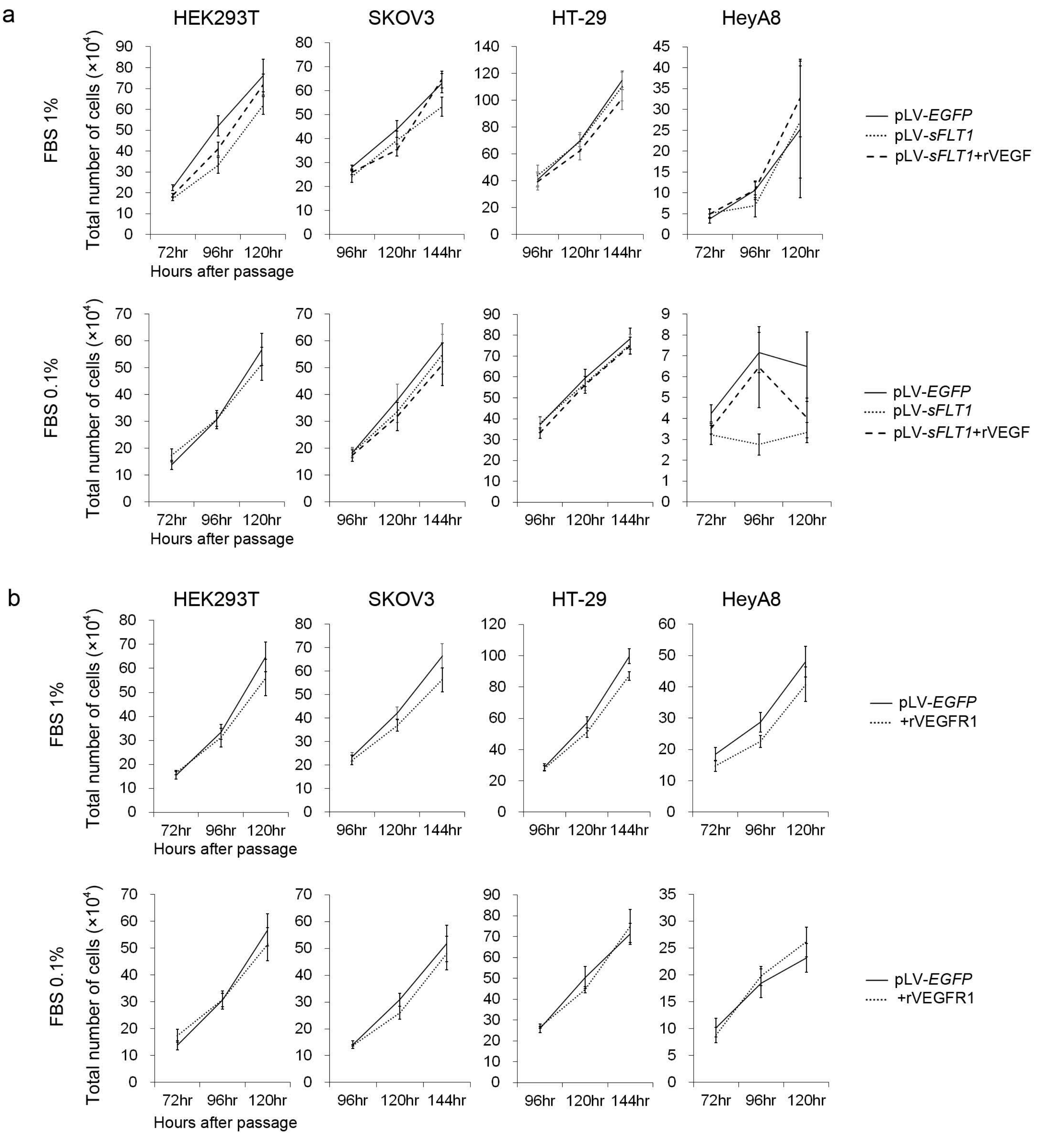

Supplementary Figure S2

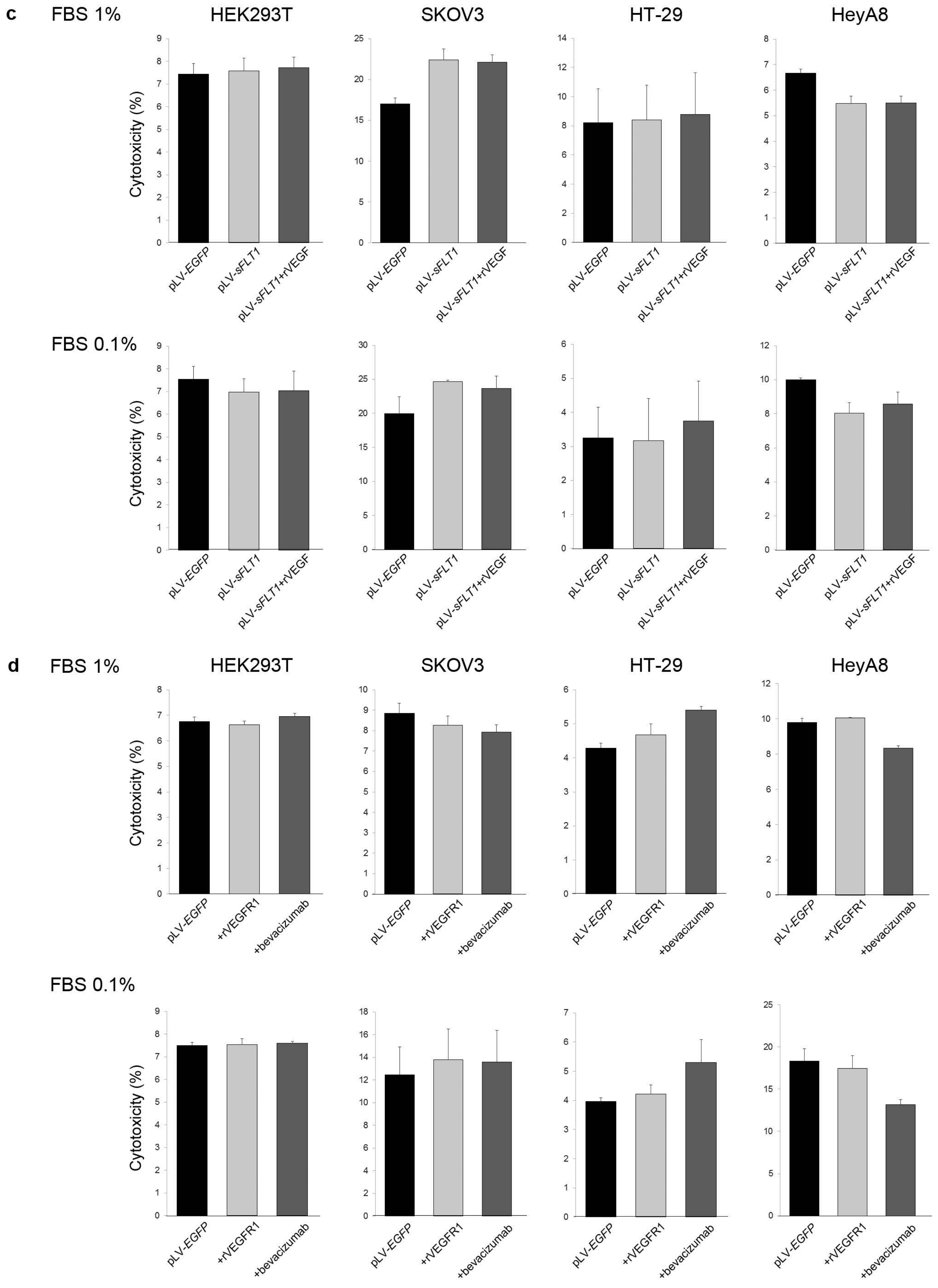

Supplementary Figure S3

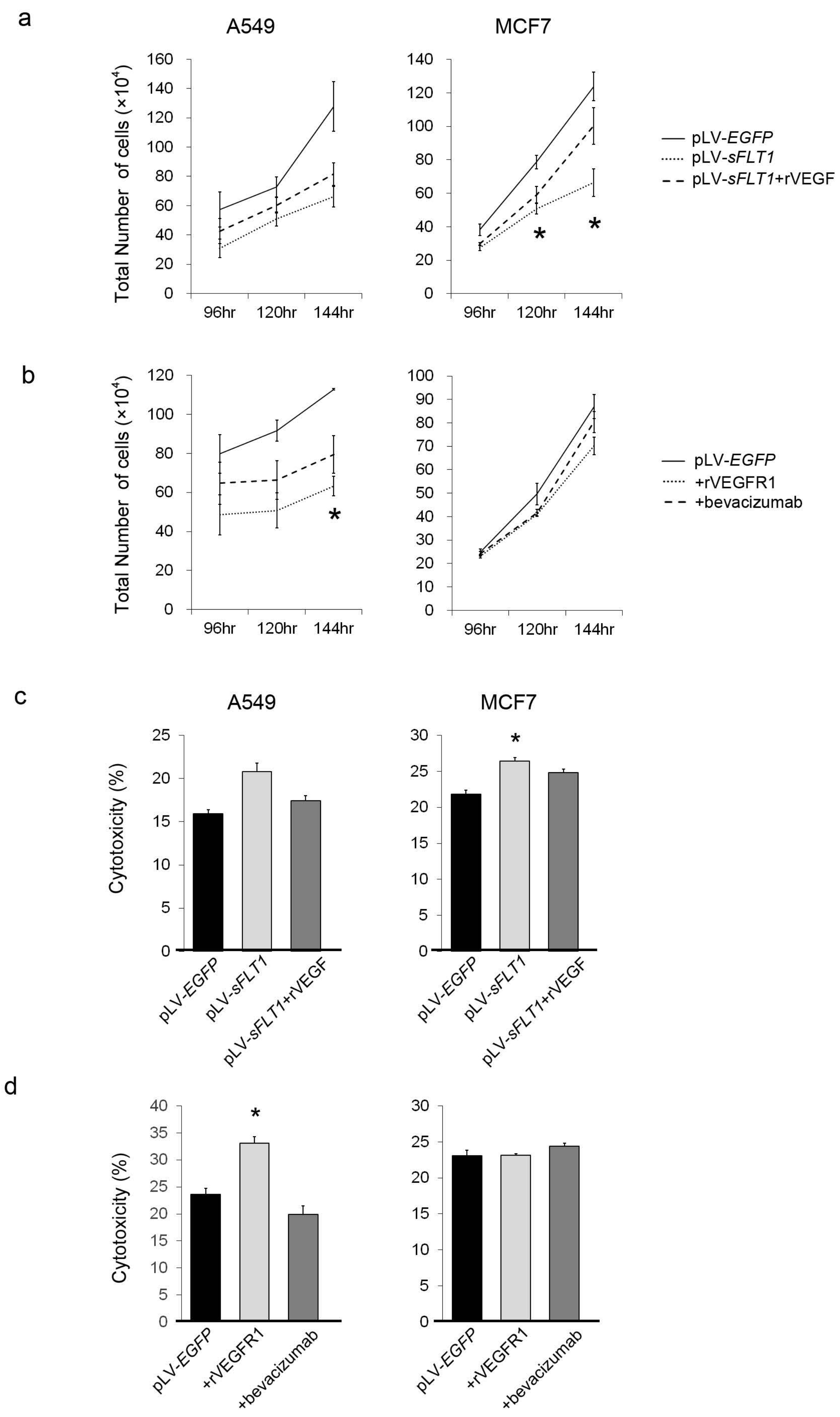

Supplementary Figure S4

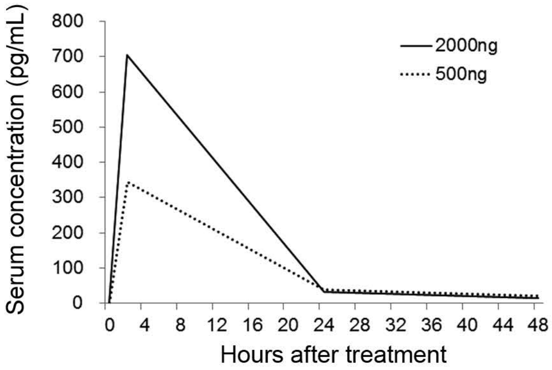

Supplementary Figure S5

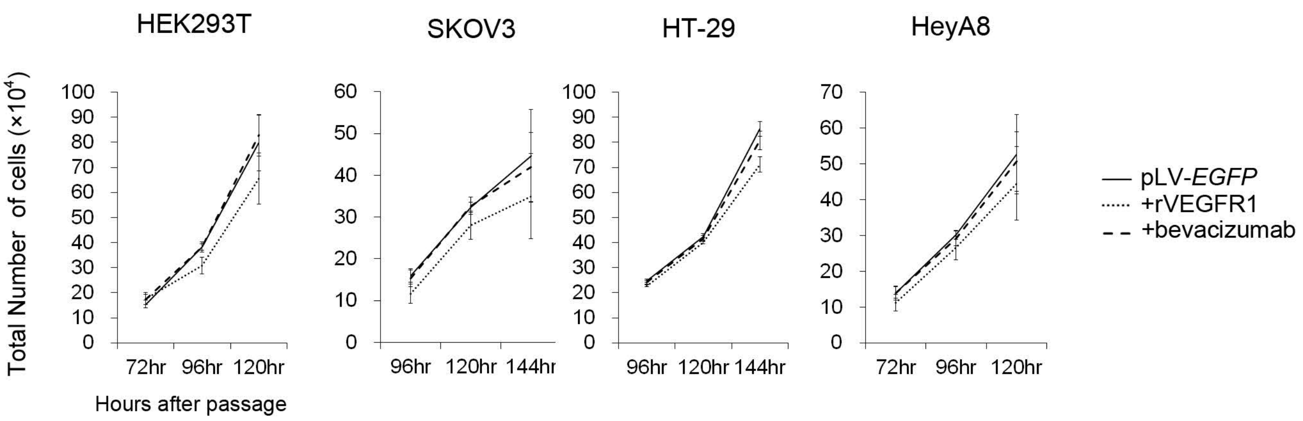

Supplementary Figure S6

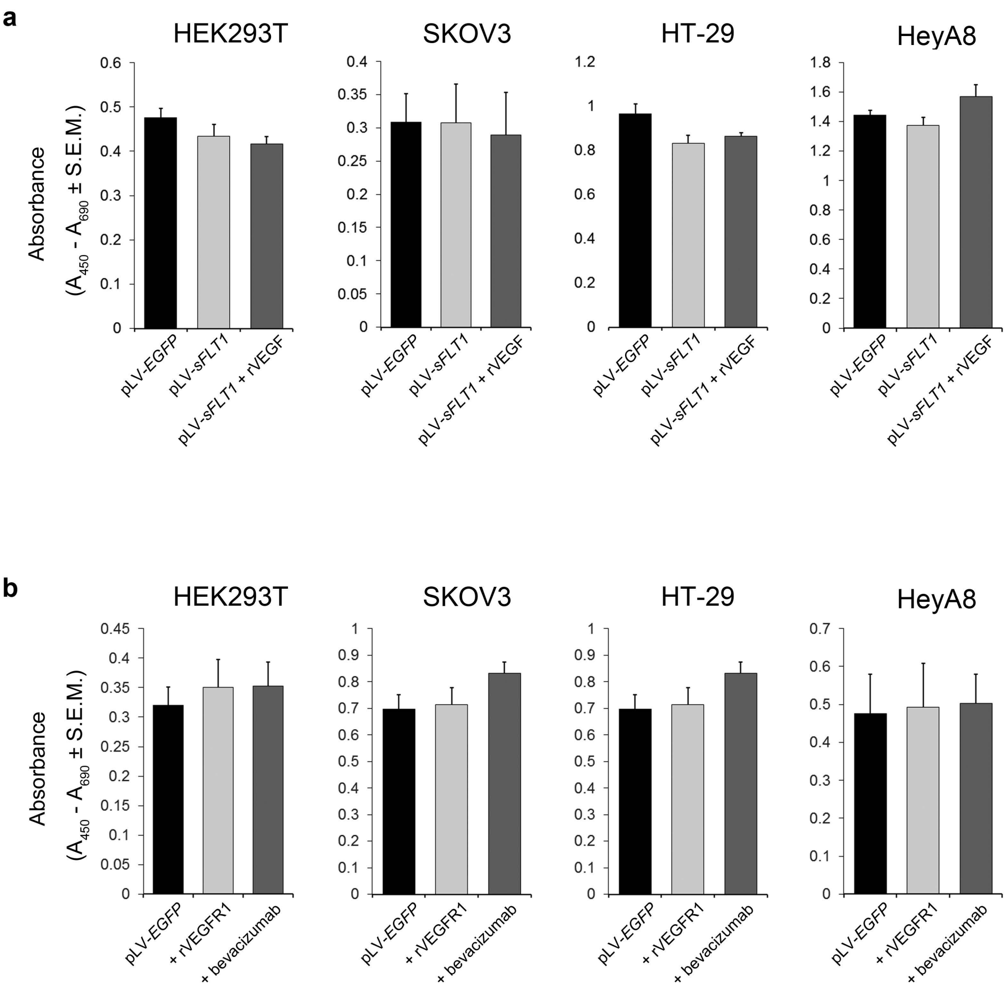

Supplementary Figure S7

a

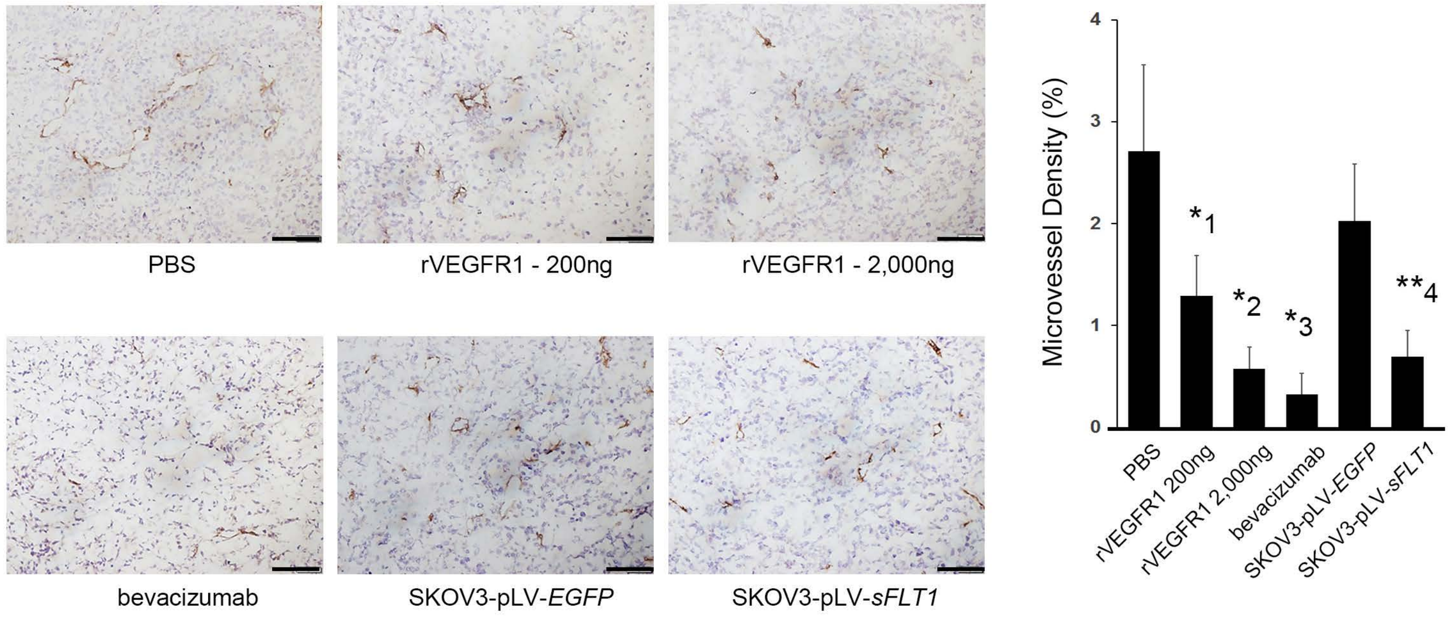

b

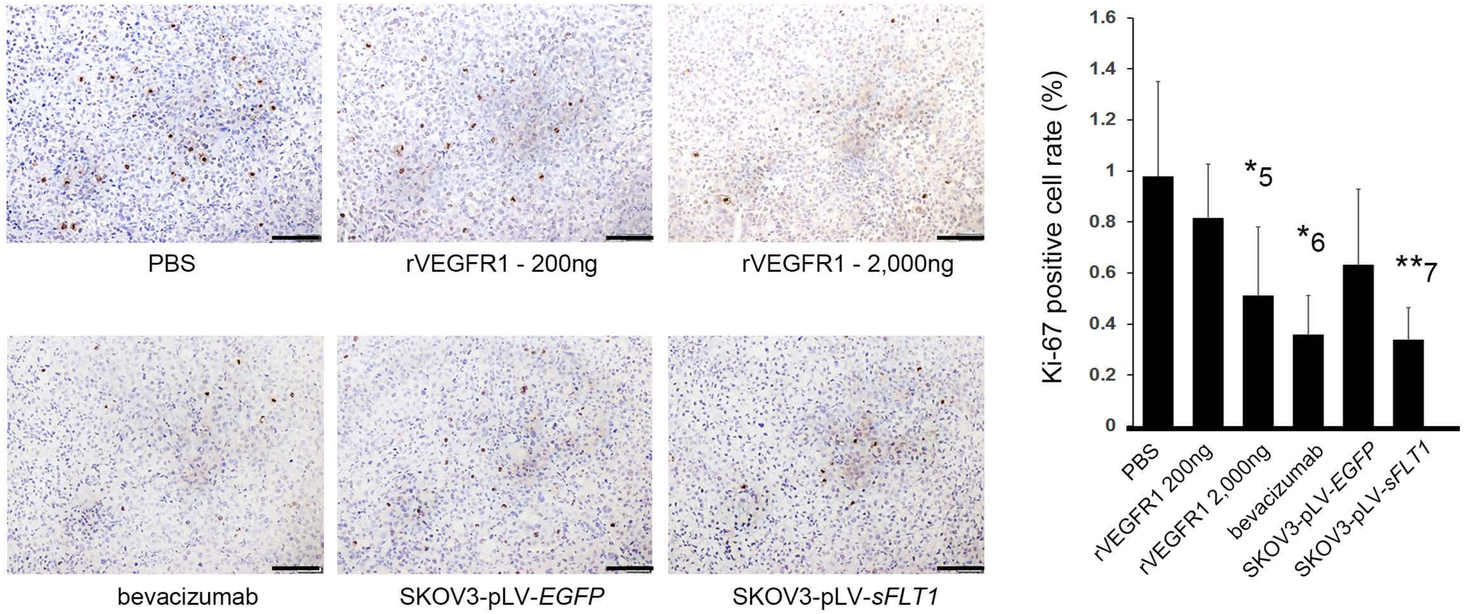

Supplementary Table S1

(pg/ml)

	pLV-*sFLT1* (ng)			
	500	250	100	50
Concentration of sFLT1	1949.66	2073.92	237.47	307.89

Supplementary Table S2

pg/ml (± S.E.)

	HEK293T			SKOV3			HT-29			HeyA8		
	pLV-*EGFP*	pLV-*sFLT1*	pLV-*sFLT1* +rVEGF	pLV-*EGFP*	pLV-*sFLT1*	pLV-*sFLT1* +rVEGF	pLV-*EGFP*	pLV-*sFLT1*	pLV-*sFLT1* +rVEGF	pLV-*EGFP*	pLV-*sFLT1*	pLV-*sFLT1* +rVEGF
sFLT1	13.14 (±0.77)	307.89 (±27.97)	333.23 (±15.70)	5.55 (±0.34)	87.52 (±24.12)	92.58 (±24.99)	5.79 (±0.38)	11.78 (±2.36)	9.51 (±1.27)	15.97 (±5.76)	21.57 (±4.20)	9.18 (±2.24)
VEGF	25.9 (±1.03)	0.11 (±0.07)	1.02 (±0.33)	344.12 (±25.14)	126.18 (±37.42)	123.36 (±39.28)	270.64 (±19.17)	245.15 (±21.63)	259.83 (±15.00)	77.88 (±3.87)	85.46 (±10.10)	109.09 (±22.03)
PlGF	11.25 (±3.89)	1.15 (±0.33)	3.79 (±0.48)	3.80 (±1.13)	4.99 (±2.71)	0.71 (±0.41)	0.00	0.00	0.43 (±0.25)	17.18 (±0.64)	27.32 (±3.20)	19.43 (±2.05)
Cell number (×10^4)	33.3 (±0.9)	13.8 (±0.9)	16.7 (±0.9)	38.1 (±3.2)	18.9 (±2.3)	23.9 (±1.8)	76.6 (±2.8)	43.1 (±1.6)	57.5 (±3.3)	38.3 (±2.4)	29.4 (±1.1)	33.0 (±1.7)

pg/ml (± S.E.)

	HEK293T			SKOV3			HT-29			HeyA8		
	pLV-*EGFP*	pLV-*EGFP* +rVEGFR1	pLV-*EGFP* +bev.	pLV-*EGFP*	pLV-*EGFP* +rVEGFR1	pLV-*EGFP* +bev.	pLV-*EGFP*	pLV-*EGFP* +rVEGFR1	pLV-*EGFP* +bev.	pLV-*EGFP*	pLV-*EGFP* +rVEGFR1	pLV-*EGFP* +bev.
sFLT1	47.78 (±0.42)	69.77 (±1.20)	45.63 (±0.27)	4.28 (±0.57)	49.98 (±0.40)	9.98 (±0.65)	5.92 (±0.11)	36.4 (±5.24)	8.78 (±0.96)	5.10 (±0.31)	28.06 (±3.93)	5.78 (±0.42)
VEGF	52.31 (±1.42)	34.84 (±0.45)	1.01 (±0.25)	446.45 (±22.06)	318.89 (±14.07)	0.68 (±0.20)	282.16 (±15.74)	246.80 (±14.14)	0.95 (±0.28)	113.55 (±21.31)	100.81 (±25.70)	0.34 (±0.19)
PlGF	8.86 (±0.28)	4.72 (±0.87)	4.34 (±0.74)	0.00	0.00	0.20 (±0.12)	0.02 (±0.01)	0.58 (±0.33)	0.36 (±0.21)	28.31 (±6.04)	31.34 (±8.21)	26.12 (±7.85)
Cell number (×10^4)	38.3 (±1.3)	30.8 (±3.4)	38.2 (±2.1)	32.3 (±1.7)	28.0 (±1.2)	32.7 (±1.0)	42.3 (±1.3)	40.0 (±0.5)	41.7 (±1.2)	34.5 (±0.7)	23.8 (±0.5)	33.6 (±3.4)

SupplementaryTable S3

a

(Average ± S.E.)
(mmHg)

	s-BP	d-BP	MBP	P value (MBP against PBS group)
PBS	98.5 ± 2.7	61.8 ± 3.4	73.8 ± 2.9	-
rVEGFR1 200ng	100.1 ± 2.6	65.7 ± 2.3	76.9 ± 2.3	0.44
rVEGFR1 2,000ng	99.5 ± 3.4	60.6 ± 2.2	76.9 ± 1.6	0.90
bevacizumab	98.1 ± 2.7	62.9 ± 1.0	73.4 ± 2.1	0.89

	s-BP	d-BP	MBP	P value (MBP against SKOV3-pLV-*EGFP* group)
SKOV3-pLV-*EGFP*	98.7 ± 1.8	56.7 ± 2.3	69.9 ± 1.4	-
SKOV3-pLV-*sFLT1*	102.2 ± 3.9	61.6 ± 3.1	75.1 ± 2.9	0.16

b

(Average ± S.E.)

	UAlb/UCr	P value (against PBS group)
PBS	4.89 ± 1.83	-
rVEGFR1 200ng	5.64 ± 0.28	0.56
rVEGFR1 2,000ng	5.87 ± 1.20	0.40
bevacizumab	4.36 ± 1.10	0.64

	UAlb/UCr	P value (against SKOV3-pLV-*EGFP* group)
SKOV3-pLV-*EGFP*	3.59 ± 1.35	-
SKOV3-pLV-*sFLT1*	3.68 ± 1.11	0.92
